# International Physicians Delphi Survey: Managing Patients With IgA Nephropathy

**DOI:** 10.1016/j.ekir.2022.05.022

**Published:** 2022-05-26

**Authors:** Jürgen Floege, Jonathan Barratt, Rosanna Coppo, Richard Lafayette, Jai Radhakrishnan, Heather N. Reich, Brad H. Rovin, David T. Selewski, Marina Vivarelli, Christopher Pham, Vladimír Tesař

**Affiliations:** 1 Division of Nephrology and Immunology, Rheinisch-Westfälische Technische Hochschule Aachen, Aachen, Germany; 2 Department of Cardiovascular Sciences, University of Leicester, Leicester, UK; 3 Fondazione Ricerca Molinette, Regina Margherita Hospital, Turin, Italy; 4 Department of Nephrology, Stanford University, Stanford, California, USA; 5 Division of Nephrology, Columbia University Medical Center, New York, New York, USA; 6 Division of Nephrology, Department of Medicine, University Health Network and University of Toronto, Toronto, Ontario, Canada; 7 Division of Nephrology, Department of Internal Medicine, The Ohio State University Wexner Medical Center, Columbus, Ohio, USA; 8 Division of Pediatric Nephrology, Department of Pediatrics, Medical University of South Carolina, Charleston, South Carolina, USA; 9 Division of Nephrology and Dialysis, Department of Pediatric Subspecialties, Bambino Gesù Pediatric Hospital Istituto di Ricerca e Cura a Carattere Scientifico, Rome, Italy; 10 ApotheCom, San Francisco, California, USA; 11 Department of Nephrology, Charles University and General University Hospital, Prague, Czech Republic

**Keywords:** corticosteroids, Delphi, guidelines, IgA nephropathy, IgAN, proteinuria

## Introduction

IgA nephropathy (IgAN) typically is a slowly progressing disease, with 10% to 60% of patients developing chronic kidney failure within 10 years.^1^ Practice guidelines (e.g., Kidney Disease: Improving Global Outcomes [KDIGO]) are available, but it is unknown how uniformly nephrologists in different countries agree with and may follow these guidelines. The Delphi Focal Segmental Glomerulosclerosis and IgA Nephropathy Experts: Physicians (DEFINE: Physicians) study sought to capture nephrologist opinions on IgAN pathophysiology, diagnosis, treatment, and monitoring. In this 2-round Delphi survey, agreement with 20 statements about IgAN was scored by adult and pediatric nephrologists from 7 countries using a 1 to 9 Likert scale (9 = strongly agree). Moderate versus high consensus was defined as 75% to 89% versus ≥90% of participants scoring 7 to 9, respectively. Methods and participant characteristics are detailed in the Supplementary Materials.

In round 1, most statements (19 [95%]) met the criteria for high consensus, including those regarding pathophysiology, diagnosis, and treatment of early stage and rapidly progressive IgAN (Table 1). Participants agreed that proteinuria is a key determinant of prognosis and treatment strategy. High levels of agreement were also observed for statements regarding the use of angiotensin-converting enzyme inhibitors or angiotensin II receptor blockers as part of supportive therapy in patients with persistent proteinuria (96%, 98%; number [#]27 and #30, respectively; Table 1), corticosteroid use in pediatric patients (98%; #31; Table 1), and treatment of rapidly progressive or severe disease with corticosteroids and cyclophosphamide in appropriate patients (97%, 94%; #29 and #32, respectively; Table 1).

**Table 1:** Statements with high consensus in round 1

#	Statement	Round 1 results			
Statements rated by all participants		n	% Agree	Median	Mean (SD)
1	Persistently elevated proteinuria is a major adverse prognostic marker in FSGS and IgAN.	207	97	9	8.43 (0.96)
2	Damage to podocytes and other glomerular cells are amplified by activation of angiotensin and/or endothelin pathways, contributing to high levels of proteinuria and greater risk for progressive kidney injury.	207	92	8	7.96 (1.07)
3	Persistent proteinuria causes tubulointerstitial injury by inducing and amplifying inflammation, fibrosis, and kidney scarring, thereby driving further disease progression.	207	98	8	8.22 (0.82)
4	A close correlation exists between the level of proteinuria and the risk of kidney failure; the higher the proteinuria, the higher the risk of kidney failure.	207	96	9	8.22 (1.14)
23	IgAN without an associated condition is considered primary or idiopathic. IgAN associated with another condition (e.g., IgA vasculitis, chronic liver disease) is defined as secondary.	207	94	8	7.88 (1.34)
**Statements rated by adult nephrologists only**					
19	In both FSGS and IgAN, the goal of therapy is to reduce proteinuria as much as safely possible to preserve kidney function as evidenced by stable or improved GFR.	157	98	9	8.34 (0.87)
24	Proteinuria determines prognosis. Patients with proteinuria >1 g/d despite optimized supportive care are at high risk for progressive kidney dysfunction or kidney failure and should be considered for more aggressive treatment.	157	94	8	7.92 (0.96)
25	The International IgAN Prediction Tool should be used at time of kidney biopsy to identify adult patients with a poor prognosis or high risk for kidney failure within 5 yr.	156^a^	90	8	7.73 (1.09)
27	ACE-I/ARBs are used as first-line maintenance treatment in patients with persistent proteinuria, except in circumstances of very advanced disease (e.g., GFR <20 ml/min per 1.73 m^2^) or acute presentation of rapidly progressive glomerulonephritis.	157	96	8	8.13 (1.13)
29	In rapidly progressive glomerulonephritis (≥50% decline in eGFR >3 mo or less), corticosteroids and cyclophosphamide are treatment options in specific settings where the risk-benefit profile is acceptable.	157	97	8	8.07 (0.91)
33	Monitoring proteinuria every 6–12 mo is recommended for patients with proteinuria <0.5 g/d and normal GFR.	157	95	8	8.08 (1.05)
34	Those with proteinuria >0.5 g/d should be monitored more frequently than 6–12 mo and monitoring should be individualized on a case-by-case basis.	157	96	8	8.07 (1.09)
**Statements rated by pediatric nephrologists only**					
26	Proteinuria determines prognosis. Patients with proteinuria >0.5 g/d per 1.73 m^2^ or urine PCR >500 mg/g despite optimized supportive care are at high risk for progressive kidney dysfunction or kidney failure and should be considered for more aggressive treatment.	50	98	8	8.06 (0.91)
30	ACE-I or ARBs are used as first-line maintenance treatment in children with persistent proteinuria.	50	98	8	8.16 (0.87)
31	If proteinuria levels cannot be reduced to <0.5 g/d per 1.73 m^2^ or to urinary PCR <500 mg/g with a course of supportive therapy using ACE-Is or ARBs, corticosteroids may be considered in specific settings where the risk-benefit profile is acceptable.	50	98	8	7.84 (0.84)
32	Children with severe disease on biopsy (severe mesangial and endocapillary hypercellularity or with crescent formations involving >30% of the glomeruli) can be treated with steroid pulses and cyclophosphamide shortly after kidney biopsy.	50	94	8	7.70 (1.25)
35	The goal of therapy is to prevent kidney damage by reducing proteinuria as much as possible to attain long-term complete remission, which is defined as the disappearance of hematuria, accompanied by proteinuria <200 mg/d per 1.73 m^2^ or PCR <200 mg/g and eGFR within normal range for age.	50	92	8	7.86 (1.28)
36	Stable disease in remission is defined as proteinuria <200 mg/d per 1.73 m^2^ or PCR <200 mg/g and with eGFR persistently in the normal range for the patient’s age.	50	98	8	8.00 (0.83)
37	Those with stable disease in remission are monitored at least once every 3–6 mo, whereas those with progressive disease should be monitored more frequently and monitoring should be individualized based on disease severity and treatment.	50	100	8	8.38 (0.67)

#, number; ACE-I, angiotensin-converting enzyme inhibitor; ARB, angiotensin II receptor blocker; eGFR, estimated glomerular filtration rate; FSGS, focal segmental glomerulosclerosis; GFR, glomerular filtration rate; IgAN, IgA nephropathy; PCR, protein-to-creatinine ratio.

Consensus was defined as median and mean agreement rating of ≥7 and ≥75% of participants rating agreement (i.e., 7–9). Statements with ≥90% agreement were considered to have reached high consensus.

a One participant indicated “I do not know” in response to this statement and was excluded from the analysis.

The only statement not meeting criteria for high consensus in round 1 was statement #28, which described short-term use of corticosteroids in adult patients with high levels of proteinuria despite optimized supportive therapy with a renin-angiotensin-aldosterone system inhibitor (Table 2). With 89% of participants agreeing, this statement met criteria for moderate consensus. The percentage of adult nephrologists agreeing with this statement was significantly lower than the percentage of pediatric nephrologists agreeing with statement #31, which addresses corticosteroid use in pediatric patients (89% vs. 98%; *P* = 0.044). For round 2, statement #28 was split into 2 statements (#28A and #28B; Table 2). The first statement, regarding corticosteroid use in patients with high proteinuria despite optimal supportive care (#28A), was rated separately from the statement that corticosteroids should not be used long term (#28B). Levels of agreement with the revised statements in round 2 were very similar to round 1 results (88%, 89%; #28A and #28B, respectively) and remained at a moderate consensus level.

**Table 2:** Statements with moderate consensus in round 1 that were retested in round 2

#	Statement	Round 1 results				Round 2 results			
Statements rated by adult nephrologists only		n	% Agree	Median	Mean (SD)	n	% Agree	Median	Mean (SD)
28	If proteinuria levels cannot be reduced to <1 g/d with a 3–6-mo course of supportive therapy using ACE-I or ARBs, a short-term 6-mo course of corticosteroids may be considered in specific settings where the risk-benefit profile is acceptable. Corticosteroids are not used as long-term maintenance therapy.	157	89	8	7.69 (1.24)	126	88	8	7.85 (1.08)
28A	If proteinuria levels cannot be reduced to <1 g/d with a 3–6-mo course of supportive therapy using ACE-I or ARBs, a short-term 6-mo course of corticosteroids may be considered in specific settings where the risk-benefit profile is acceptable.	Not tested				126	88	8	7.87 (1.05)
28B	Corticosteroids are not used as long-term maintenance therapy.	Not tested				126	89	8	7.98 (1.30)

#, number; ACE-I, angiotensin-converting enzyme inhibitor; ARB, angiotensin II receptor blocker.

Further analyses explored differences in agreement levels among participants practicing in different geographic locations (Supplementary Tables S1 and S2)*.* When comparing responses from North American versus European participants, most statements had similar levels of agreement, but in round 1, statement #28 on corticosteroid use in adults had a >10% difference (Supplementary Table S1; 95% of North American nephrologists vs. 82% of European nephrologists agreed; *P* = 0.011). Further analyses by country can be found in Supplementary Table S2. In addition, there were no significant differences between academic and nonacademic nephrologists in how both groups rated statements, except for statement #28. This statement had 83% agreement among academic nephrologists and 93% agreement among nonacademic nephrologists (Supplementary Table S3; *P* = 0.045). In round 2, the difference in agreement between academic and nonacademic nephrologists for both statements #28A and #28B was no longer statistically significant (*P* = 0.460 and 0.486, respectively). It is possible that academic nephrologists may assess the long-term risk-benefit profile of corticosteroids more conservatively than their nonacademic colleagues.

The DEFINE: Physicians Delphi survey on IgAN identified high consensus overall after just 1 round of the survey. This finding suggests that nephrologists in North America and Europe have similar opinions on IgAN management and that these opinions are largely consistent with the KDIGO guideline. The statement with the lowest level of agreement (although still moderate) was statement #28, which described corticosteroid use in adults. Corticosteroids for managing IgAN is a controversial topic, considering recent results from studies including the STOP-IGAN and TESTING trials.^2^, ^3^, ^4^, ^5^ Results from these trials reveal that although there is some evidence for corticosteroid efficacy in particular in Asian patients, they come with significant risks for adverse events.

The high level of agreement found for the pathophysiology and treatment goal statements (Table 1; #1–4, #19, and #35) reveals that nephrologists see proteinuria as an important prognostic marker and that reducing proteinuria as much as possible is vital to preserving kidney function. Most nephrologists agreed that proteinuria itself is also a driver of disease progression by causing or contributing to kidney damage (Table 1; #3).^6^

Most statements in this survey aligned with the 2021 KDIGO guideline for glomerular diseases.^7^ However, some slight differences exist in specific laboratory values for initiating the use of renin-angiotensin-aldosterone system inhibitors in adult patients. The KDIGO guideline suggests starting renin-angiotensin-aldosterone system inhibitors if proteinuria is >0.5 g/d, but statement #27 for this survey did not mention a specific proteinuria value. In pediatric patients, the 2021 KDIGO guideline has no statement that directly parallels statement #31 in our survey, which describes initiating corticosteroids in children if proteinuria remains >0.5 g/d despite supportive therapy. Last, the 2021 KDIGO guideline does not provide specific information on monitoring or follow-up frequency,^7^ whereas statements #33, #34, and #37 from this study do address this topic (Table 1).

The high levels of agreement found in this study may be driven by the fact that treatment options for IgAN are limited, with no alternatives to consider. However, the treatment landscape of IgAN may change, as many studies are currently investigating potential therapies for this disease.^8^ For instance, a delayed-release budesonide formulation was recently approved by the Food and Drug Administration for IgAN treatment.^9^

Limitations of this study include that it was conducted in English and did not involve nephrologists from Asia or South America. Furthermore, there was a limited number of female participants and pediatric nephrologists in this survey (Supplementary Tables S4 and S5). Statements were written by the research team and steering committee (Supplementary Table S6) and were thus predetermined before the survey was administered. To ensure high completion and retention rates, this survey investigated a limited number of statements. Statements on experimental therapies or therapies with little evidence available may have resulted in lower consensus. Furthermore, some statements in this survey combined several points or topics, which may have affected agreement if a participant disagreed with one part but not all of the statement. Another limitation is attrition bias; participants who did not return in round 2 potentially had different perspectives than those who responded in round 2 (Supplementary Tables S7 and S8).

In summary, the lowest levels of agreement were observed on corticosteroid use in adult patients who receive optimized supportive therapy but still have elevated proteinuria. Although overall agreement was high regarding this topic, some nephrologists, particularly in Europe, disagreed with the use of corticosteroids in this setting. This suggests that further research on the risk-benefit profile of corticosteroids and new therapies in IgAN are needed. Overall, the DEFINE: Physicians study found high levels of consensus regarding the pathophysiology, diagnosis, management, and monitoring of IgAN among nephrologists from North America and Europe and that, in general, their opinions align with the latest KDIGO guideline for statements evaluated herein.

## Disclosure

JF is employed by Rheinisch-Westfälische Technische Hochschule University of Aachen; has consultancy agreements with Amgen, Bayer, Calliditas, Novo Nordisk, Omeros, Travere Therapeutics, Inc., Vifor, and Visterra; has received honoraria from Amgen, Astellas, Bayer, Calliditas, Novo Nordisk, Omeros, Travere Therapeutics, Inc., Vifor, and Visterra; is a scientific advisor for Calliditas, Omeros, and Travere Therapeutics, Inc.; and is on the speakers bureau for Amgen and Vifor. JB has received research grants from Argenx, Calliditas, Chinook Therapeutics, Galapagos, GSK, Novartis, Travere Therapeutics, Inc., and Vera Therapeutics; and serves as a medical/scientific advisor to Alnylam Pharmaceuticals, Argenx, Astellas, Biocryst, Calliditas, Chinook Therapeutics, Dimerix, Galapagos, GlaxoSmithKline, Novartis, Omeros, Travere Therapeutics, Inc., UCB, Vera Therapeutics, and Visterra. RC has consultancy agreements with Amgen, Argenx, Calliditas, Novartis, Ostuka, Reata, Recordati, and Travere Therapeutics, Inc., and has an agreement with UpToDate. RL has received research funding from Calliditas, Chinook, National Institutes of Health, Novartis, Travere Therapeutics, Inc., Pfizer, Vera, Omeros, and Visterra; and is an advisor for Alexion, Calliditas, Chemocentryx, Chinook, Novartis Omeros, Pfizer, Reatta, Travere Therapeutics, Inc., and Visterra. JR has received research grants from Travere Therapeutics, Inc.; is on a steering committee for Travere Therapeutics, Inc.; and has consulting/advisory board roles with Angion Biomedica and Travere Therapeutics, Inc. HNR has received consulting fees from Calliditas, Chinook, Novartis, and Travere Therapeutics, Inc.; has received honoraria from Novartis; is an advisor for Novartis and Travere Therapeutics, Inc.; has served as national coordinating investigator for trials by Calliditas and Chinook; has served as an investigator for GN clinical trials by Alnylam, Calliditas, Chemocentryx, Omeros, and Pfizer; and is director of the Glomerulonephritis Fellowship funded by the Louise Fast Foundation. BR has received consulting fees from Calliditas, Novartis, Omeros, and Travere Therapeutics, Inc. DTS has consultancy agreements with BioPorto and Travere Therapeutics, Inc. MV is on advisory boards for Apellis, Novartis, Roche, and Travere Therapeutics, Inc.; receives consulting fees from Alexion; and has participated in studies sponsored by Bayer, Novartis, Chemocentrix, and Chinook. This does not influence the content of the present study. CP is employed by ApotheCom, which received funding support from Travere Therapeutics, Inc. for the DEFINE: Physicians study. VT has served as principal investigator and steering committee member for clinical studies in focal segmental glomerulosclerosis supported by Travere Therapeutics, Inc., and has consultancy agreements with AstraZeneca, Boehringer Ingelheim, Calliditas, Novartis, Omeros, and Travere Therapeutics, Inc.

## Supplementary Material

**Supplementary Methods.**

**Figure S1.** Participant disposition.

**Table S1.** Analysis of select statements in rounds 1 and 2 by geographic region.

**Table S2.** Analysis of select statements by country.

**Table S3.** Analysis of select statements by practice setting.

**Table S4.** Characteristics used for participant screening.

**Table S5.** Additional participant characteristics.

**Table S6.** Steering committee and research team membership.

**Table S7.** Participant countries and specialties: round 1 and round 2.

**Table S8.** Comparison of characteristics between participants and nonparticipants in round 2.
