# Supplementary material for: International Physicians Delphi Survey: Managing Patients With IgA Nephropathy
Source: Kidney Int Rep. 2022 May 26;7(9):2076–80. doi: 10.1016/j.ekir.2022.05.022 (PMC9458983; doi:10.1016/j.ekir.2022.05.022)
Supplement: Supplementary File (PDF) [file mmc1.pdf]

Supplementary Materials

International Physicians Delphi Survey on Managing Patients With IgA Nephropathy

Authors: Jürgen Floege, Jonathan Barratt, Rosanna Coppo, Richard Lafayette,
Jai Radhakrishnan, Heather N. Reich, Brad H. Rovin, David T. Selewski, Marina Vivarelli,
Christopher Pham, Vladimir Tesař

Supplementary Materials Table of Contents

	**Page**
Supplementary Materials and Methods	2
Patient Characteristics	5
Impact of Geographic Location by Country	5
Supplementary Figure S1. Participant disposition.	6
Supplementary Table S1. Analysis of select statements in rounds 1 and 2 by geographic region.	7
Supplementary Table S2. Analysis of select statements by country.	8
Supplementary Table S3. Analysis of select statements by practice setting.	11
Supplementary Table S4. Characteristics used for participant screening.	12
Supplementary Table S5. Additional participant characteristics.	14
Supplementary Table S6. Steering committee and research team membership.	17
Supplementary Table S7. Participant countries and specialties: round 1 and round 2.	18
Supplementary Table S8. Comparison of characteristics between participants and nonparticipants in round 2.	19

Supplementary Materials and Methods

Study Design

An online Delphi method was used to explore nephrologist opinions and to gain consensus on statements describing immunoglobulin A nephropathy (IgAN) pathophysiology, diagnosis, treatment, and monitoring. Participants were nephrologists in North America and Europe who had experience diagnosing and managing patients with IgAN (Supplementary Table S4).

The study involved 2 rounds of an online survey sent to participants. In round 1, participants were presented with 20 statements on IgAN management and kidney disease pathophysiology (Tables 1 and 2). Participants were asked to rate each statement using a 1–9 Likert scale (1 = strongly disagree, 9 = strongly agree) or to check "I do not know." Agreement with a statement was defined as rating 7–9. Participants were required to provide feedback for any statement rated <7. Consensus was defined as meeting all 3 of the following criteria: mean rating ≥7, median rating ≥7, and ≥75% of participants rating a statement as 7–9 (agreement). Moderate consensus was defined as 75% to 89% agreement; high consensus was defined as ≥90% agreement.

Statements not meeting criteria for high consensus in round 1 were revised based on participant feedback and retested in round 2. In addition to rating revised statements in round 2, participants were asked to again rate the original statement based on the results of round 1, taking into consideration their previous rating, the overall rating distribution from all participants, the median rating, and the percentage of participants who agreed with the statement in round 1.

Statement Development and Revisions

The study design and protocol were developed and approved by the steering committee, which was comprised of IgAN experts (Supplementary Table S6). A research team of nephrologists with expertise in managing IgAN in adult and pediatric patients (Supplementary Table S6) developed the initial statements based on areas of relevance identified by the steering

committee and, partially, the 2021 Kidney Disease: Improving Global Outcomes guideline for glomerular diseases.[S1] Nephrologist opinions on pathophysiology, diagnosis, treatment, and monitoring were deemed as areas worthy of further understanding. The research team developed statements in these areas of interest based on existing clinical guidelines, literature reviews, and their clinical experience. Statements on pathophysiology were to be rated by all participants, whereas statements on managing IgAN in adult or pediatric patients were rated only by participants with the appropriate clinical experience. Statements in round 2 were revised by the research team and approved by the steering committee.

Study Population

Participants were board-certified nephrologists from Canada, France, Germany, Italy, Spain, the United Kingdom (UK), and the United States (US); had practiced nephrology for ≥5 years; and had diagnosed or treated ≥2 patients with IgAN in the previous 2 years. The protocol was planned to include pediatric nephrologists as approximately 30% of all participants. To ensure that opinions of nephrologists practicing in nonacademic and community settings were represented, the proportion of nephrologists who treat adults in academic centers was limited to <40% of participants. This limit was not imposed on pediatric nephrologists or on any participants from the UK (all nephrologists from the UK were expected to work in academic settings).

A market research firm (Psyma International Inc., Berwyn, PA, USA, and affiliated partners) assisted in recruitment using validated lists of nephrologists updated through sources such as the American Medical Association and the European Medical Association, hospital books/directories, medical directories, and verified healthcare internet sites. Participants were compensated for completing the surveys.

Ethics Review

The Biomedical Research Alliance of New York reviewed the study protocol and gave an Exempt Status applicable in the US. Institutional review board (IRB) statements confirming that IRB approval was not necessary were collected from non-US countries where the survey was administered. Informed consent was obtained from each participant.

Data Collection and Analysis

Demographics and other characteristics were collected in round 1. Descriptive statistics, including mean, median, and percentages, were used to summarize statement ratings collected from each round. The one recorded "I do not know" response was excluded from analysis. Statistical analyses were conducted using Microsoft Excel and SPSS Version 28.[S2] Percentages of agreement between round 1 and round 2 were compared using McNemar's test. Agreement levels based on participants' clinical work settings and geographic location were compared using Pearson's chi-squared tests. All tests used a significance threshold of $P = 0.05$.

Participant Characteristics

Round 1 of the survey was administered between November 17, 2020, and January 14, 2021, with 207 nephrologists participating (Supplementary Figure S1). Nephrologist characteristics are detailed in Supplementary Table S4. Round 2 was administered between March 29, 2021, and April 13, 2021, with 126 of 157 (80%) nephrologists who treat adults and 32 of 50 (64%) nephrologists who treat children participating again (Supplementary Figure S1; Supplementary Tables S7 and S8). Overall, 76% of patients from round 1 participated in round 2.

Impact of Geographic Location by Country

Further analysis of Statement #28 by country identified that participants' agreement in continental European countries was 86% to 100%, whereas only 40% of participants from the UK agreed (Supplementary Table S2; $P < 0.001$). In round 2, agreement with revised Statement #28A ranged from 67% to 100% in all countries (Supplementary Table S2; $P = 0.216$). For Statement #28A, UK nephrologists' agreement was at 80%, whereas Spanish nephrologists' agreement was at 67%. For Statement #28B, agreement ranged from 75% (Italy) to 100% (Canada). Overall, agreement among North American and European nephrologists was high for all statements and all statements met consensus criteria.

References

S1. Kidney Disease: Improving Global Outcomes (KDIGO) CKD Work Group. KDIGO 2021 clinical practice guideline for the management of glomerular diseases. Kidney Int. 2021;100(45):S1-S276.

S2. IBM SPSS Statistics for Windows VA, NY: IBM Corp.

Supplementary Figure S1. Participant disposition.

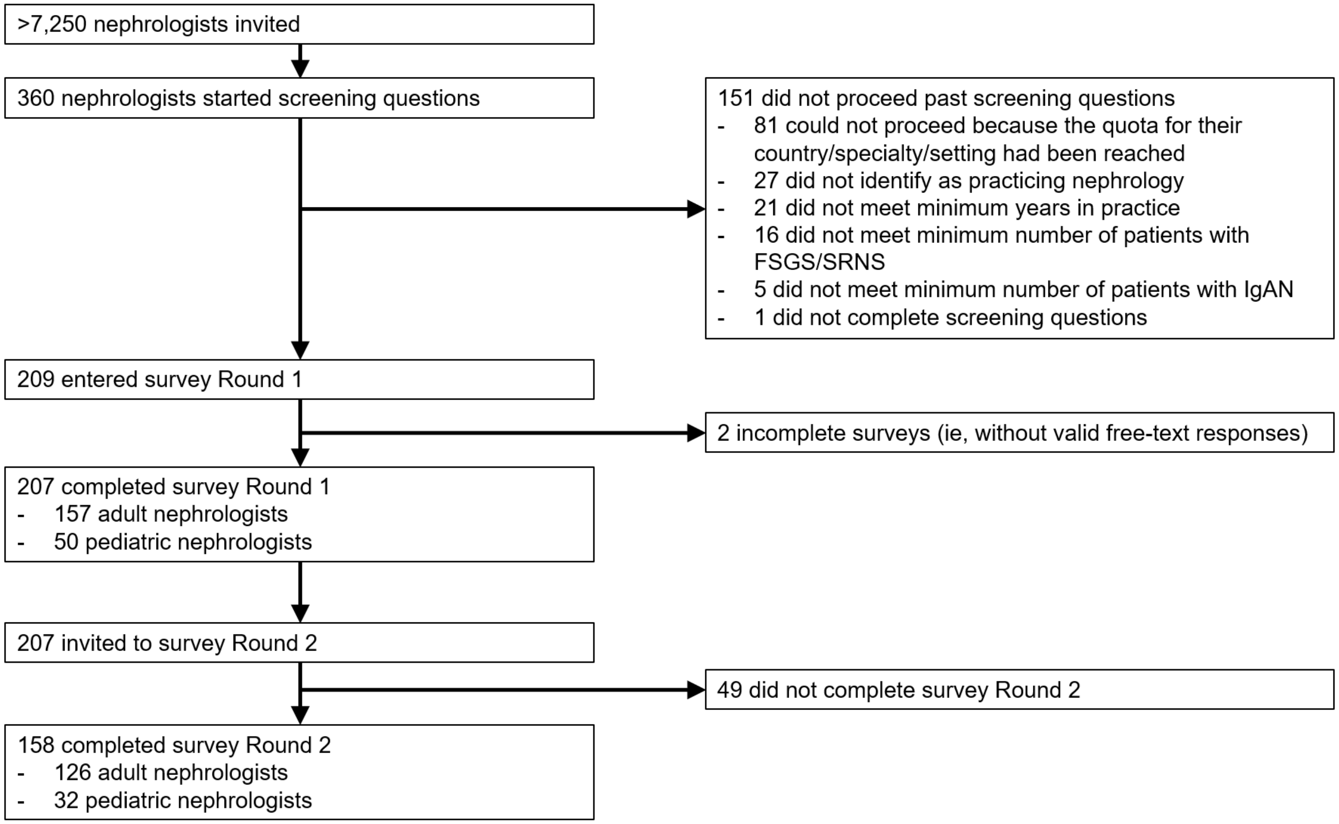

Note that this survey involved both FSGS and IgAN questions, and thus some potential participants were excluded from the survey due to lack of experience in treating FSGS/SRNS. Only results regarding the IgAN questions are reported in this paper; results for FSGS questions are reported in a separate paper.

FSGS, focal segmental glomerulosclerosis; IgAN, IgA nephropathy; SRNS, steroid-resistant nephrotic syndrome.

Supplementary Table S1. Analysis of select statements[a] in rounds 1 and 2 by geographic region.

#	Statement	North America				Europe			
**Statements rated by adult nephrologists only**		***n***	**% Agree**	**Median**	**Mean (SD)**	***n***	**% Agree**	**Median**	**Mean (SD)**
28	If proteinuria levels cannot be reduced to <1 g/day with a 3–6-month course of supportive therapy using ACE-I or ARBs, a short-term 6-month course of corticosteroids may be considered in specific settings where the risk-benefit profile is acceptable. Corticosteroids are not used as long-term maintenance therapy.	79	95%	8	7.95 (1.01)	78	82%	8	7.44 (1.39)
28A	If proteinuria levels cannot be reduced to <1 g/day with a 3–6-month course of supportive therapy using ACE-I or ARBs, a short-term 6-month course of corticosteroids may be considered in specific settings where the risk-benefit profile is acceptable.	69	94%	8	8.04 (0.93)	57	81%	8	7.65 (1.16)
28B	Corticosteroids are not used as long-term maintenance therapy.	69	93%	8	8.03 (1.31)	57	84%	8	7.93 (1.29)

ACE-I, angiotensin-converting enzyme inhibitor; ARB, angiotensin II receptor blocker.

[a]Statements with a statistically significant ≥10% difference in agreement between North America and Europe in round 1.

Supplementary Table S2. Analysis of select statements[a] by country.

#	Statement				
**Statements rated by adult nephrologists only**					
28	If proteinuria levels cannot be reduced to <1 g/day with a 3–6-month course of supportive therapy using ACE-I or ARBs, a short-term 6-month course of corticosteroids may be considered in specific settings where the risk-benefit profile is acceptable. Corticosteroids are not used as long-term maintenance therapy.	***n***	**% Agree**	**Median**	**Mean (SD)**
	Canada	10	90%	8	7.90 (1.29)
	France	17	100%	8	7.94 (0.83)
	Germany	14	86%	7	7.50 (1.22)
	Italy	19	89%	8	7.79 (0.85)
	Spain	13	92%	8	7.92 (1.04)

	United Kingdom	15	40%	6	5.93 (1.87)
	United States	69	96%	8	7.96 (0.98)
28A	If proteinuria levels cannot be reduced to <1 g/day with a 3–6-month course of supportive therapy using ACE-I or ARBs, a short-term 6-month course of corticosteroids may be considered in specific settings where the risk-benefit profile is acceptable.	***n***	**% Agree**	**Median**	**Mean (SD)**
	Canada	8	100%	8.5	8.50 (0.53)
	France	12	83%	8	7.92 (1.16)
	Germany	10	90%	8	7.90 (1.10)
	Italy	16	81%	8	7.63 (1.20)
	Spain	9	67%	8	7.44 (1.51)
	United Kingdom	10	80%	7.5	7.30 (0.82)

	United States	61	93%	8	7.98 (0.96)
28B	Corticosteroids are not used as long-term maintenance therapy.	***n***	**% Agree**	**Median**	**Mean (SD)**
	Canada	8	100%	9	8.50 (0.76)
	France	12	92%	8	8.08 (1.00)
	Germany	10	90%	9	8.60 (0.97)
	Italy	16	75%	8	7.44 (1.63)
	Spain	9	89%	8	7.78 (0.97)
	United Kingdom	10	80%	8.5	8.00 (1.41)
	United States	61	92%	8	7.97 (1.35)

ACE-I, angiotensin-converting enzyme inhibitor; ARB, angiotensin II receptor blocker.

[a]Statements with a ≥10% difference in agreement between North America and Europe in round 1 and its round 2 revisions.

Supplementary Table S3. Analysis of select statements[a] by practice setting.

#	Statement	Academic				Nonacademic			
**Statements rated by adult nephrologists only**		***n***	**% Agree**	**Median**	**Mean (SD)**	***n***	**% Agree**	**Median**	**Mean (SD)**
28	If proteinuria levels cannot be reduced to <1 g/day with a 3–6-month course of supportive therapy using ACE-I or ARBs, a short-term 6-month course of corticosteroids may be considered in specific settings where the risk-benefit profile is acceptable. Corticosteroids are not used as long-term maintenance therapy.	70	83%	8	7.46 (1.34)	87	93%	8	7.89 (1.13)
28A	If proteinuria levels cannot be reduced to <1 g/day with a 3–6-month course of supportive therapy using ACE-I or ARBs, a short-term 6-month course of corticosteroids may be considered in specific settings where the risk-benefit profile is acceptable.	56	86%	8	7.70 (1.09)	70	90%	8	8.00 (1.01)
28B	Corticosteroids are not used as long-term maintenance therapy.	56	91%	8	8.07 (1.25)	70	87%	8	7.91 (1.34)

ACE-I, angiotensin-converting enzyme inhibitor; ARB, angiotensin II receptor blocker.

[a]Statements with a ≥10% difference in agreement between academic and nonacademic nephrologists in round 1.

Supplementary Table S4. Characteristics used for participant screening.

Characteristics	Adult Nephrologists (*n* = 157)	Pediatric Nephrologists (*n* = 50)	All (*N* = 207)
Experience in years, median (range)	18 (5–49)	17 (5–40)	18 (5–49)
No. of patients with IgAN diagnosed and/or treated in the last 2 years			
Median	25	20	25
Mean	42.8	48.3	44.2
Range	2–400	2–385	2–400
IQR	11–50	10–70	11–50
Practice setting, ***n*** (%)			
Academic center or academic hospital	70 (45)	34 (68)	104 (50)
Nonacademic	87 (55)	16 (32)	103 (50)
Community hospital (non-teaching)	37 (24)	7 (14)	44 (21)
Private office	49 (31)	8 (16)	57 (28)
Other	1 (1)	1 (2)	2 (1)
Country of practice, ***n*** (%)			
Canada	10 (6)	1 (2)	11 (5)
France	17 (11)	2 (4)	19 (9)
Germany	14 (9)	7 (14)	21 (10)
Italy	19 (12)	4 (8)	23 (11)
Spain	13 (8)	5 (10)	18 (9)
United Kingdom	15 (10)	5 (10)	20 (10)
United States	69 (44)	26 (52)	95 (46)
**Other characteristics of interest**			

Principal investigator in clinical trials,[a] ***n*** (%)	33 (21)	16 (32)	49 (24)
No. of trials, median (range)	1 (1–5)	2 (1–5)	1 (1–5)
Author/coauthor on nephrology publications in the past 5 years, ***n*** (%)	90 (57)	36 (72)	126 (61)
No. of publications, median (range)	4.5 (1–165)	4 (1–80)	4 (1–165)
Participants referencing ≥1 clinical guideline when treating patients,[b] ***n*** (%)	144 (92)	47 (94)	191 (92)
KDIGO	138 (88)	33 (66)	171 (83)
IPNA	7 (4)	27 (54)	34 (16)
ERKNet	17 (11)	13 (26)	30 (14)
AAFP	4 (3)	5 (10)	9 (4)
Other	13 (8)	5 (10)	18 (9)

AAFP, American Academy of Family Physicians; ERKNet, The European Rare Kidney Disease Reference Network; FSGS, focal segmental glomerulosclerosis; IgAN, IgA nephropathy; IPNA, International Pediatric Nephrology Association; IQR, interquartile range; KDIGO, Kidney Disease – Improving Global Outcomes.

[a]Limited to clinical trials enrolling patients with FSGS or IgAN in the last 5 years.

[b]Participants were able to select all that apply.

Supplementary Table S5. Additional participant characteristics.

Characteristic	Adult nephrologists *n* = 157	Pediatric nephrologists *n* = 50	All *N* = 207
Gender, ***n*** (%)			
Male	130 (83)	40 (80)	170 (82)
Female	25 (16)	8 (16)	33 (16)
Nonbinary	1 (1)	0	1 (<1)
Not disclosed	1 (1)	2 (4)	3 (1)
Ethnic background (US participants only),[a] ***n*** (%)	*n* = 69	*n* = 26	*n* = 95
White or Caucasian	34 (49)	18 (69)	52 (55)
Asian	26 (38)	7 (27)	33 (35)
Multiracial	2 (3)	0	2 (2)
Black or African American	1 (1)	0	1 (1)
Latin American or Hispanic	0	0	0
Native American or Alaska Native	0	0	0
Native Hawaiian or other Pacific Islander	0	0	0
Other	3 (4)	0	3 (3)
Not disclosed	3 (4)	1 (4)	4 (4)
Presenter at ≥1 congress attended by nephrologists,[a] ***n*** (%)	88 (56)	26 (52)	114 (55)
ASN	43 (27)	14 (28)	57 (28)
ERA–EDTA	36 (23)	11 (22)	47 (23)
NKF	12 (8)	3 (6)	15 (7)

APSN	3 (2)	3 (6)	6 (3)
ANZSN	2 (1)	1 (2)	3 (1)
ISPOR Annual International Meeting	1 (1)	1 (2)	2 (1)
ISPOR Annual European Congress	0	1 (2)	1 (<1)
ISPOR Latin America Conference	0	1 (2)	1 (<1)
AMCP Nexus	0	1 (2)	1 (<1)
Spring Managed Care Forum (NAMCP, AAIHDS, AAMCN)	0	0	0
AMCP Managed Care & Specialty Pharmacy Annual Meeting	0	0	0
Other	31 (20)	9 (18)	40 (19)
Sources of nephrology-related information used,[a] ***n*** (%)			
Articles on UpToDate	122 (78)	31 (62)	153 (74)
Nephrology journals	114 (73)	29 (58)	143 (69)
Nephrology congress attendance	104 (66)	32 (64)	136 (66)
Discussions with other nephrologists	97 (62)	38 (76)	135 (65)
Pharma representatives	44 (28)	13 (26)	57 (28)
Newsletters from nephrology societies	32 (20)	5 (10)	37 (18)
Other	7 (4)	0	7 (3)
Coverage of nephrology-related drug costs covered for most patients, ***n*** (%)			
Public insurance (government insurance or subsidized)	117 (75)	31 (62)	148 (71)
Private insurance (commercial, private, employer, and state health insurance marketplace coverage)	39 (25)	19 (38)	58 (28)

Uninsured or underinsured (and therefore out of pocket)	1 (<1)	0	1 (<1)

AAIHDS, American Association of Integrated Healthcare Delivery Systems; AAMCN, American Association of Managed Care Nurses; AMCP, Academy of Managed Care Pharmacy; ANZSN, Australian and New Zealand Society of Nephrology; APSN, Asian Pacific Society of Nephrology; ASN, American Society of Nephrology; ERA–EDTA, European Renal Association–European Dialysis and Transplant Association; ISPOR, International Society for Pharmacoeconomics and Outcomes Research; NAMCP, National Association of Managed Care Physicians; NKF, National Kidney Foundation; US, United States.

[a]Participants were able to select all that apply.

Supplementary Table S6. Steering committee and research team membership.

Name	Affiliation
**Steering Committee**	
Jürgen Floege (chair)	Division of Nephrology and Immunology, Rheinisch-Westfälische Technische Hochschule Aachen, Aachen, Germany
Jai Radhakrishnan	Division of Nephrology, Columbia University Medical Center, New York, NY, USA
Heather N. Reich	Division of Nephrology, Department of Medicine, University Health Network and University of Toronto, Toronto, ON, Canada
Vladimír Tesař	Department of Nephrology, Charles University, Prague, Czech Republic
Marcello Tonelli	Research Office of the Vice-President, Cumming School of Medicine, University of Calgary, Calgary, AB, Canada
Marina Vivarelli	Division of Nephrology and Dialysis, Department of Pediatric Subspecialties, Bambino Gesù Pediatric Hospital Istituto di Ricerca e Cura a Carattere Scientifico (IRCCS), Rome, Italy
**Research Team**	
Jonathan Barratt	University of Leicester, Leicester, UK
Rosanna Coppo	Fondazione Ricerca Molinette, Regina Margherita Hospital, Turin, Italy
Richard Lafayette	Stanford University, Stanford, CA, USA
Brad H. Rovin	The Ohio State University Wexner Medical Center, Columbus, OH, USA
David T. Selewski	Medical University of South Carolina, Charleston, SC, USA

Supplementary Table S7. Participant countries and specialties: round 1 and round 2.

Country	Specialty	Round 1	Round 2	Percentage of returning participants
Canada	Adult nephrology	10	8	80
	Pediatric nephrology	1	1	100
France	Adult nephrology	17	12	71
	Pediatric nephrology	2	2	100
Germany	Adult nephrology	14	10	71
	Pediatric nephrology	7	6	86
Italy	Adult nephrology	19	16	84
	Pediatric nephrology	4	3	75
Spain	Adult nephrology	13	9	69
	Pediatric nephrology	5	2	40
United Kingdom	Adult nephrology	15	10	67
	Pediatric nephrology	5	3	60
United States	Adult nephrology	69	61	88
	Pediatric nephrology	26	15	58
All countries	Adult nephrology	157	126	80
	Pediatric nephrology	50	32	64
Total		207	158	76

Supplementary Table S8. Comparison of characteristics between participants and nonparticipants in round 2.

Characteristics	Adult nephrologists, *n* = 157			Pediatric nephrologists,[a] *n* = 50			All, *N* = 207		
	Round 2 participant *n* = 126	Round 2 nonparticipant *n* = 31	*P* value	Round 2 participant *n* = 32	Round 2 nonparticipant *n* = 18	*P* value	Round 2 participant *n* = 158	Round 2 nonparticipant *n* = 49	*P* value
**Characteristics used for participant screening**									
Experience as a practicing nephrologist in years, median (range)	18.5 (5–38)	17 (6–49)	0.533[b]	15 (5–40)	23 (5–38)	0.155[b]	18 (5–40)	18 (5–49)	0.217[b]
No. of patients with IgAN diagnosed and/or treated in the last 2 years									
Median (IQR)	25 (11.25–50)	25 (11.5–50)	0.993[c]	24.5 (10.75–71)	20 (10.75–64)	0.911[c]	25 (11–50)	20 (11–50)	0.872[c]
Mean	43.53	40.03		48.94	47.28		44.63	42.69	
Practice setting, ***n*** (%)									
Academic center or academic hospital	56 (44)	14 (45)	0.943[d]	19 (59)	15 (83)	0.081[d]	75 (47)	29 (59)	0.152[d]

Nonacademic	70 (56)	17 (55)		13 (41)	3 (17)		83 (53)	20 (41)	
Country of practice, ***n*** (%)									
Canada	8 (6)	2 (6)	0.276[d]	1 (3)	0	0.536[d]	9 (6)	2 (4)	0.519[d]
France	12 (10)	5 (16)		2 (6)	0		14 (9)	5 (10)	
Germany	10 (8)	4 (13)		6 (19)	1 (6)		16 (10)	5 (10)	
Italy	16 (13)	3 (10)		3 (9)	1 (6)		19 (12)	4 (8)	
Spain	9 (7)	4 (13)		2 (6)	3 (17)		11 (7)	7 (14)	
United Kingdom	10 (8)	5 (16)		3 (9)	2 (11)		13 (8)	7 (14)	
United States	61 (48)	8 (26)		15 (47)	11 (61)		76 (48)	19 (39)	
**Other characteristics of interest**									
Principal investigator in clinical trials,[e] ***n*** (%)	22 (17)	11 (35)	0.027[d]	11 (34)	5 (28)	0.631[d]	33 (21)	16 (33)	0.090[d]
Author/coauthor on nephrology publications in the past 5 years, ***n*** (%)	66 (52)	24 (77)	0.012[d]	22 (69)	14 (78)	0.495	88 (56)	38 (78)	0.006[d]
Participants referencing at least 1 clinical guideline when treating patients, ***n*** (%)	114 (90)	30 (97)	0.254[d]	29 (91)	18 (100)	0.180[d]	143 (91)	48 (98)	0.088[d]

FSGS, focal segmental glomerulosclerosis; IgAN, IgA nephropathy; IQR, interquartile range.

[a]Initially, participants were asked to self-identify their specialty (adult nephrology [patients aged ≥18 years] vs. pediatric nephrology [patients aged 1–18 years]). To address recruitment challenges, this metric was later changed so that participants with ≥30% pediatric patients were considered qualified to respond to pediatric statements.

[b]*P* values calculated using Student's *t*-test.

[c]*P* values calculated using Mann-Whitney *U* test.

[d]*P* values calculated using Pearson's chi-squared test.

[e]Limited to clinical trials enrolling patients with FSGS or IgAN in the last 5 years.
